# Changes in conditional net survival and dynamic prognostic factors in patients with newly diagnosed metastatic prostate cancer initially treated with androgen deprivation therapy

**DOI:** 10.1002/cam4.2502

**Published:** 2019-09-11

**Authors:** Shintaro Narita, Kyoko Nomura, Shingo Hatakeyama, Masahiro Takahashi, Toshihiko Sakurai, Sadafumi Kawamura, Senji Hoshi, Masanori Ishida, Toshiaki Kawaguchi, Shigeto Ishidoya, Jiro Shimoda, Hiromi Sato, Koji Mitsuzuka, Tatsuo Tochigi, Norihiko Tsuchiya, Chikara Ohyama, Yoichi Arai, Kengo Nagashima, Tomonori Habuchi

**Affiliations:** ^1^ Department of Urology Akita University School of Medicine Akita Japan; ^2^ Department of Public Health Akita University School of Medicine Akita Japan; ^3^ Department of Urology Hirosaki University School of Medicine Hirosaki Japan; ^4^ Department of Urology Tohoku University School of Medicine Sendai Japan; ^5^ Department of Urology Yamagata University School of Medicine Yamagata Japan; ^6^ Department of Urology Miyagi Cancer Center Natori Japan; ^7^ Department of Urology Yamagata Prefectural Central Hospital Yamagata Japan; ^8^ Department of Urology Iwate Prefectural Isawa Hospital Mizusawa Japan; ^9^ Department of Urology Aomori Prefectural Central Hospital Aomori Japan; ^10^ Department of Urology Sendai City Hospital Sendai Japan; ^11^ Research Center for Medical and Health Data Science The Institute of Statistical Mathematics Minato‐ku Japan; ^12^ Michinoku Japan Urological Cancer Study Group (MJUCSG) Minato‐ku Japan

**Keywords:** androgen deprivation therapy, conditional survival, metastatic hormone‐naive, net survival, prostate cancer

## Abstract

**Background:**

The purpose of this study was to identify predictive factors associated with conditional net survival in patients with metastatic hormone‐naive prostate cancer (mHNPC) initially treated with androgen deprivation therapy (ADT).

**Methods:**

At nine hospitals in Tohoku, Japan, the medical records of 605 consecutive patients with mHNPC who initially received ADT were retrospectively reviewed. The Pohar Perme estimator was used to calculate conditional net cancer‐specific survival (CSS) and overall survival (OS) for up to 5 years subsequent to the diagnosis. Using multiple imputation, proportional hazard ratios for conditional CSS and OS were calculated with adjusted Cox regression models.

**Results:**

During a median follow up of 2.95 years, 208 patients died, of which 169 died due to progressive prostate cancer. At baseline, the 5‐year CSS and OS rates were 65.5% and 58.2%, respectively. Conditional 5‐year net CSS and OS survival gradually increased for all the patients. In patients given a 5‐year survivorship, the conditional 5‐year net CSS and OS rates improved to 0.906 and 0.811, respectively. Only the extent of disease score (EOD) ≥2 remained a prognostic factor for CSS and OS up to 5 years; as survival time increased, other variables were no longer independent prognostic factors.

**Conclusions:**

The conditional 5‐year net CSS and OS in patients with mHNPC gradually increased; thus, the risk of mortality decreased with increasing survival. The patient's risk profile changed over time. EOD remained an independent prognostic factor for CSS and OS after 5‐year follow‐up. Conditional net survival can play a role in clinical decision‐making, providing intriguing information for cancer survivors.

## Graphical abstract

The conditional 5‐year net CSS and OS in patients with mHNPC gradually increased. EOD remained an independent prognostic factor for CSS and OS after 5‐year follow‐up. Conditional net survival can play a role in clinical decision‐making, providing intriguing information for cancer survivors.

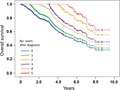

## 1 INTRODUCTION

Prostate cancer is the most common malignancy in men and the sixth leading cause of cancer‐related death worldwide.^1^ The widespread application of prostate‐specific antigen (PSA) screening has resulted in an increase in the identification of early stage prostate cancer and a reduction of metastatic prostate cancer; in Western counties, metastatic prostate cancer is found in approximately 4% of prostate cancer patients at the time of diagnosis.^2^, ^3^, ^4^ Newly diagnosed metastatic prostate cancer is generally an aggressive disease; conventional androgen deprivation therapy (ADT)‐resistant cancer (known as castration‐resistant prostate cancer) can develop, eventually proving lethal. However, patients with metastatic prostate cancer form a very heterogeneous population, with considerable variation in the response, adverse events, and clinical outcomes.^2^

Recently, large randomized trials have demonstrated a significant benefit to the overall survival (OS) of patients with metastatic hormone‐naïve prostate cancer (mHNPC) from the administration of additional upfront docetaxel and abiraterone acetate treatment.^5^, ^6^, ^7^ The treatment strategy for patients with newly diagnosed mHNPC has changed in recent years. Thus, an accurate assessment of prognosis is critical for clinical decision‐making and for providing information to patients with newly diagnosed mHNPC.

Previous studies have reported survival outcomes for patients with mHNPC that were estimated at the time of diagnosis or initial treatment.^8^, ^9^, ^10^ However, the risk of death changes over time, so the survival probability for patients who have survived for several years may change, and cancer‐specific survival (CSS) and OS rates may not be sufficiently informative for these patients. Conditional survival, which assesses the changing hazard rate as survival time increases,^11^, ^12^ provides a dynamic risk assessment and more accurate survival information for patients who have already survived for several years. Conditional survival analysis has been applied to assess the prognosis for a number of cancers, including metastatic cancers^13^, ^14^; however, only a small number of studies have investigated conditional survival for prostate cancer, especially metastatic prostate cancer.^15^, ^16^ Net survival, which measures the survival that would be observed if the only possible cause of death was the disease of interest, provides the most appropriate method of estimating survival from cancer.^17^, ^18^ The newly developed Pohar Perme estimator has been shown to provide unbiased net survival estimates that are more accurate than classical relative survival estimates.^19^ However, there is little evidence regarding estimates of conditional survival using an unbiased Pohar Perme estimator in cancer populations.^20^, ^21^

In this multicenter retrospective cohort study, we evaluated changes in conditional net survival in patients with mHNPC initially treated with ADT, at time points from 1 to 5 years after the initial diagnosis, using the Pohar Perme estimator. We also evaluated the impact of potential prognostic factors on CSS and OS in this study population.

## 2 MATERIALS AND METHODS

### 2.1 Patients

This retrospective multicenter study was conducted at nine medical institutions in the Tohoku region of Japan. A consecutive group of adult patients diagnosed with mHNPC between March 2008 and May 2016 was retrospectively identified at each institute; in total, this included 629 patients. All the patients initially received ADT, which comprised orchiectomy and luteinizing hormone‐releasing agonists/antagonists alone or combined with bicalutamide. No patient received upfront docetaxel and/or abiraterone acetate as an initial therapy. Sequential treatments were administered after first‐line ADT at the physician's discretion. The study was approved by each institution's ethics committee. An opt‐out method for consent was adopted, in which patients were informed of their inclusion in the study and were provided information on the institution's website.

### 2.2 Assessment

Continuous variables for the study cohort are presented as mean ± standard deviation or as medians with interquartile ranges (IQRs), and categorical variables as counts and percentages. The variables in the data set comprised the following patient characteristics at the time of diagnosis: age; body mass index (BMI; kg/m^2^); Eastern Cooperative Oncology Group Performance Status score (ECOG‐PS); biopsy Gleason score; site of metastasis (visceral, lymph node, or bone); presence of bone pain; bone metastasis extent of disease (EOD) score; types of initial hormonal therapy; implementation of local treatment; levels of serum biomarker PSA, hemoglobin (Hb), alkaline phosphatase (ALP), lactate dehydrogenase (LDH), and date of cause‐specific death or all‐cause death. ECOG‐PS and the presence of bone pain were evaluated by inquiry and physical examination. EOD scores were classified according to the definition of Soloway et al^22^ using bone scintigraphy at the time of the initial diagnosis.

**Figure 1:**
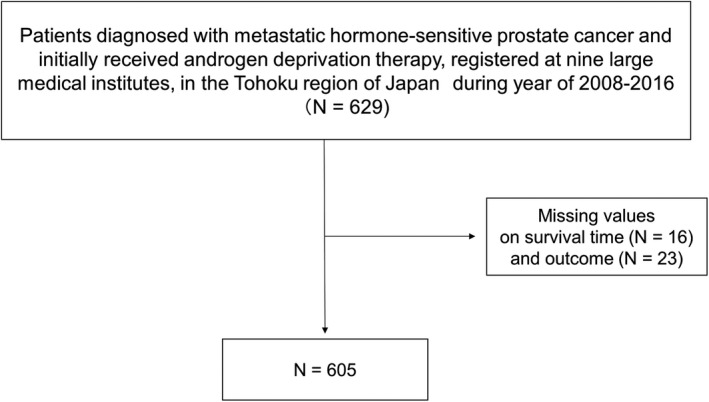
Flowchart of study enrollment

Study enrollment is summarized in Figure 1. Of the initial 629 patients, 24 were excluded because of missing values on survival outcome. The remaining 605 patients comprised the subjects in our analyses.

### 2.3 Statistical analyses

CSS and OS were calculated as the time from the diagnosis of mHNPC to death from prostate cancer or from any other cause. Patients known to be alive or lost to follow‐up on the date of last contact were censored. To estimate CSS and OS, we used conditional survival, the multiplicative probability, indicating that 5‐year conditional survival represents the probability of surviving an additional 5 years, given that the patient has already survived *x* years (where *x* is the time elapsed since the diagnosis of mHNPC). Net survival, a non‐parametric unbiased estimator, was used as a measure of conditional survival, calculated by using the “stns” command in Stata statistical software.^19^, ^23^ The Kaplan‐Meier method was applied to depict CSS and OS curves, which were compared using the log‐rank test. We applied Kaplan‐Meier survival analysis to calculate CSS and OS conditional probabilities.^12^, ^24^

Factors associated with the CSS and OS were investigated by using Cox multivariate models. Multiple imputations of missing values of covariates (PSA, Hb, ALP, LDH, and BMI, EOD score, the presence of bone pain, ECOG‐PS, Gleason score, local treatment, luteinizing hormone‐releasing treatment) by chained equations (number of imputations, 200) were performed using the “MI” procedures in SAS statistical software, assuming the mechanism of missingness at random.

In the Cox regression models, PSA levels were categorized as high or low according to whether they were above or below the median value of its distribution at baseline (297 ng/mL). The other serum biomarkers were divided into binary groups according to whether they were above or below the following median values of the normal ranges for Japanese men^25^, ^26^, ^27^: HGB (≤12 g/dL vs >12 g/dL<), ALP (>350 IU vs ≤350 IU), and LDH (>220 IU vs ≤220 IU). After entering all potential predictors for CSS and OS, we ran backward selection based on type III score^28^ chi‐square test statistics to identify the most suitable models. We then checked whether the selected variables satisfied the proportional hazard assumption and calculated the hazard ratio and 95% confidence interval (CI). In the final models, we calculated the hazard ratio and 95% CI of each covariate for conditional net survival.

Statistical analyses were performed using SPSS ver. 19.0, Stata ver. 14, and SAS ver. 9.4. A *P*‐value < .05 was considered statistically significant.

## 3 RESULTS

### 3.1 Patient characteristics

Table 1 shows the patients' characteristics. For the 605 patients analyzed, the mean age was 72 ± 8.6 years. The mean BMI and median baseline PSA level were 22.7 ± 3.6 kg/m^2^ and 295.0 ng/mL (IQR 68.1‐854.8 ng/mL), respectively. Regarding metastatic sites, 90.9%, 51.4%, and 11.6% of the patients had bone, lymph node, and visceral metastases, respectively. The percentages of patients with EOD scores of 1, 2, and ≥3 were 34.1%, 26.5%, and 30.3%, respectively. A combined androgen blockade was used for 81.6% of the patients, and 16.1% were treated using luteinizing hormone‐releasing antagonists.

**Table 1:** Patient characteristics at the time of diagnosis

Variables	n = 605
Age, y, mean (SD)	72 (8.6)
BMI, kg/m^2^, mean (SD)	22.7 (3.6)
Missing (n = 158)*	23.1 (0.5)
ECOG‐PS, n (%)	
0	333 (57.9)
≥1	242 (42.1)
Missing*	30
Biopsy Gleason score, n (%)	
≤8	256 (45.2)
≥9	311 (54.9)
Missing*	38
Site of metastasis, n (%)	
Bone	550 (90.9)
Lymph node	311 (51.4)
Visceral	70 (11.6)
Presence of bone pain, n (%)	
Yes	212 (38.3)
No	341 (61.7)
Missing*	52
EOD score, n (%)	
0	55 (9.1)
1	206 (34.1)
2	160 (26.5)
3	130 (21.5)
4	53 (8.8)
Missing*	1
Serum markers	
Baseline PSA level, ng/ml, median (IQR)	295.0 (68.1‐854.8)
Missing (n = 7)*	297.3 (68.1‐865.3)
Baseline Hb level, g/dl, median (IQR)	13.4 (11.7‐14.4)
Missing (n = 52)*	13.4 (11.7‐14.5)
Baseline ALP level, IU, median (IQR)	351.0 (248.0‐791.0)
Missing (n = 60)*	352.0 (238.0‐883.0)
Baseline LDH level, IU, median (IQR)	210.0 (179.0‐262.0)
Missing (n = 116)*	213.0 (176.0‐288.0)
Hormone therapy	
LHRH antagonist	
Yes	97 (16.1)
No	506 (83.9)
Missing*	2
Combined with antiandrogen	
Yes	492 (81.6)
No	111 (18.4)
Missing*	2
Local treatment	
Yes	36 (6.0)
No	565 (94.0)
Missing*	4

Abbreviations: ALP, alkaline phosphatase; BMI, body mass index; ECOG‐PS, Eastern Cooperative Oncology Group Performance Status; EOD, extent of bone disease; Hb, hemoglobin; IQR, Interquartile range; PSA, prostate‐specific antigen; LDH, lactate dehydrogenase; LHRH, Luteinizing hormone‐releasing hormone

* Summary statistics for variables in the imputed datasets (200 imputations).

### 3.2 Treatment outcome

During the follow‐up period (median, 2.95 years), a total of 208 patients died, 169 from prostate cancer. The 5‐year CSS and OS for all the patients were 65.5% and 58.2%, respectively. In the univariate analyses at baseline, the following were significantly associated with CSS and OS: BMI ≤18.5 kg/m^2^, ECOG‐PS ≥1, biopsy Gleason score ≥9, EOD score ≥2, low Hb level at baseline, high ALP level at baseline, and high LDH level at baseline (See Tables S1 and S2). In addition, the presence of lymph node metastasis was significantly associated with CSS, and age ≥73 years was significantly associated with OS. The multivariate analysis showed that biopsy Gleason score ≥9, EOD score ≥2, low Hb level at baseline, and high LDH level at baseline were independent prognostic factors for CSS and OS. BMI ≤18.5kg/m^2^ and ECOG‐PS ≥1 were also independent prognostic factors for OS and the presence of lymph node metastasis was an independent factor for CSS.

### 3.3 Conditional survival

**Figure 2:**
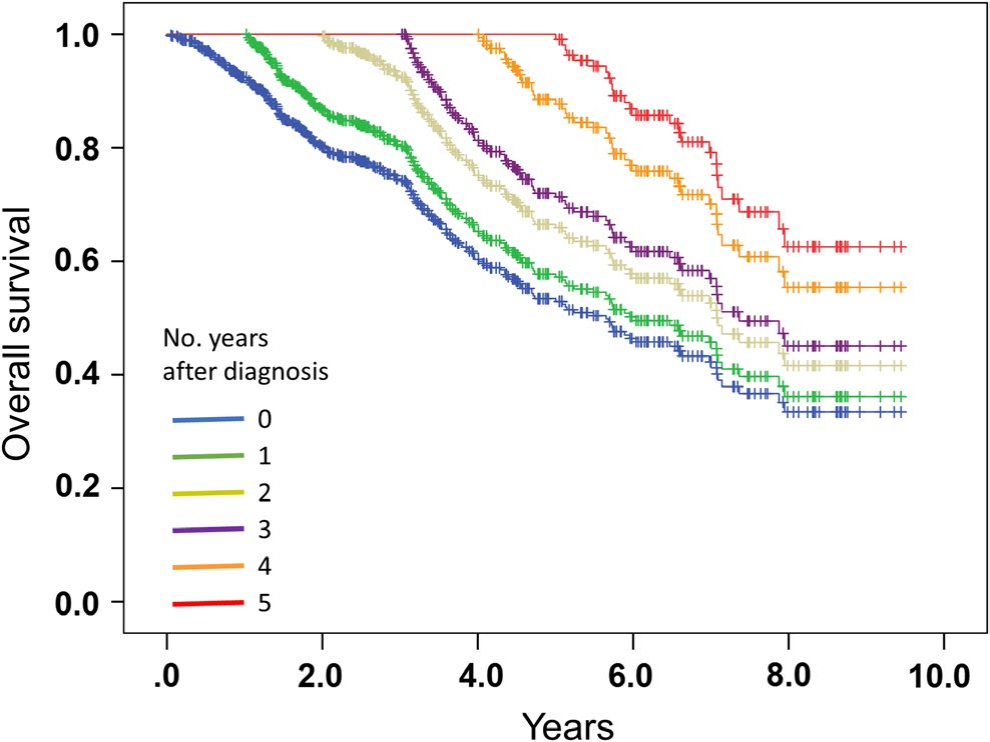
Conditional overall survival curve for patients with metastatic hormone‐naive prostate cancer initially treated with androgen deprivation therapy

Table 2 and Table S3 present the conditional 5‐year net CSS and OS rates. The overall conditional 5‐year net CSS rate at baseline was 0.656, and the overall conditional 5‐year net CSS rates (with the difference from baseline) for patients who survived for 1, 2, 3, 4, and 5 years were 0.645 (−0.11), 0.683 (+0.27), 0.652 (−0.04), 0.802 (+1.06), and 0.906 (+2.10), respectively (Table S3). The overall conditional 5‐year net OS rate at baseline was 0.582, and the overall conditional 5‐year net OS rates for patients who survived for 1, 2, 3, 4, and 5 years were 0.566 (−0.16), 0.615 (+3.3), 0.550 (−0.32), 0.702 (+1.2), and 0.811 (+2.29), respectively (Table 2). Kaplan‐Meier curves of conditional CSS and OS are shown in Figure 2 and Figure S1. These results demonstrated that conditional 5‐year CSS and OS rates gradually improved compared to baseline for at least 5 years after the initial ADT.

**Table 2:** Conditional 5‐y net overall survival of patients in relation to clinical and tumor characteristics

	Baseline	1 y	2 y	3 y	4 y	5 y
Cohort, n	605	488	341	249	165	112
5‐y CS rates	0.582 (95%CI:0.521‐0.643)	0.566 (95%CI:0.493‐0.639)	0.615 (95%CI:0.525‐0.705)	0.550 (95%CI: 0.429‐0.672)	0.702 (95%CI: 0.554‐0.851)	0.811 (95%CI: 0.648‐0.975)
Age, y						
≥73	0.548 (95%CI:0.455‐0.640)	0.573 (95%CI:0.461‐0.684)	0.660 (95%CI:0.518‐0.802)	0.686 (95%CI: 0.492‐0.879)	0.860 (95%CI:0.629‐1.091)	1.062 (95%CI: 0.803‐1.322)
<73	0.615 (95%CI:0.538‐0.693)	0.556 (95%CI:0.462‐0.649)	0.568 (95%CI: 0.455‐0.682)	0.437 (95%CI: 0.290‐0.585)	0.566 (95%CI: 0.382‐0.749)	0.611 (95%CI: 0.416‐0.805)
BMI, kg/m^2^						
<18.5	0.476 (95%CI:0.369‐0.583)	0.490 (95%CI:0.369‐0.612)	0.589 (95%CI: 0.449‐0.730)	0.596 (95%CI: 0.410‐0.782)	0.868 (95%CI: 0.636‐1.099)	0.987 (95%CI:0.750‐1.225)
18.5‐24.9	0.622 (95%CI:0.532‐0.712)	0.602 (95%CI:0.494‐0.710)	0.592 (95%CI: 0.455‐0.728)	0.504 (95%CI:0.325‐0.683)	0.600 (95%CI: 0.391‐0.810)	0.712 (95%CI: 0.474‐0.951)
>25	0.659 (95%CI:0.535‐0.782)	0.594 (95%CI:0.437‐0.751)	0.708 (95%CI: 0.531‐0.886)	0.591 (95%CI: 0.353‐0.829)	0.767 (95%CI: 0.475‐1.058)	0.839 (95%CI: 0.528‐1.150)
ECOG‐PS						
≥1	0.492 (95%CI:0.400‐0.584)	0.517 (95%CI:0.405‐0.630)	0.566 (95%CI: 0.427‐0.704)	0.541 (95%CI: 0.371‐0.711)	0.753 (95%CI: 0.533‐0.972)	0.899 (95%CI:0.655‐1.143)
0	0.677 (95%CI:0.595‐0.758)	0.622 (95%CI:0.523‐0.720)	0.673 (95%CI: 0.556‐0.790)	0.561 (95%CI: 0.386‐0.737)	0.677 (95%CI: 0.472‐0.882)	0.742 (95%CI: 0.522‐0.961)
Biopsy Gleason score						
≥9	0.522 (95%CI:0.440‐0.605)	0.478 (95%CI:0.381‐0.576)	0.547 (95%CI: 0.428‐0.665)	0.503 (95%CI: 0.333‐0.672)	0.656 (95%CI: 0.444‐0.868)	0.766 (95%CI: 0.529‐1.003)
≤8	0.703 (95%CI:0.612‐0.793)	0.709 (95%CI:0.604‐0.815)	0.723 (95%CI:0.585‐0.861)	0.601 (95%CI: 0.411‐0.790)	0.741 (95%CI: 0.517‐0.965)	N/A
Site of metstasis						
Lymph node						
Yes	0.555 (95%CI:0.472‐0.638)	0.515 (95%CI:0.413‐0.617)	0.567 (95%CI: 0.444‐0.691)	0.497 (95%CI: 0.332‐0.662)	0.605 (95%CI: 0.410‐0.800)	0.716 (95%CI: 0.495‐0.937)
No	0.609 (95%CI:0.519‐0.699)	0.623 (95%CI:0.522‐0.724)	0.667 (95%CI: 0.541‐0.793)	0.607 (95%CI: 0.435‐0.779)	0.810 (95%CI:0.596‐1.025)	0.906 (95%CI: 0.678‐1.134)
Visceral						
Yes	0.482 (95%CI:0.284‐0.681)	0.568 (95%CI:0.340‐0.797)	0.518 (95%CI: 0.211‐0.825)	0.540 (95%CI: 0.221‐0.859)	N/A	N/A
No	0.592 (95%CI:0.528‐0.657)	0.565 (95%CI:0.489‐0.642)	0.608 (95%CI:0.512‐0.704)	0.552 (95%CI: 0.422‐0.681)	0.703 (95%CI: 0.545‐0.861)	0.803 (95%CI:0.629‐0.976)
Presence of bone pain						
Yes	0.578 (95%CI:0.474‐0.682)	0.556 (95%CI:0.421‐0.690)	0.530 (95%CI: 0.360‐0.699)	0.453 (95%CI: 0.254‐0.651)	0.583 (95%CI: 0.336‐0.830)	0.647 (95%CI:0.381‐0.914)
No	0.599 (95%CI:0.516‐0.682)	0.589 (95%CI:0.494‐0.685)	0.673 (95%CI: 0.559‐0.787)	0.644 (95%CI: 0.497‐0.791)	0.821 (95%CI: 0.648‐0.994)	0.959 (95%CI: 0.773‐1.146)
EOD score						
≥2	0.466 (95%CI:0.385‐0.547)	0.429 (95%CI:0.333‐0.525)	0.433 (95%CI:0.313‐0.553)	0.300 (95%CI:0.158‐0.442)	0.394 (95%CI:0.2120.576)	0.478 (95%CI:0.263‐0.692)
≤1	0.732 (95%CI:0.645‐0.819)	0.710 (95%CI:0.612‐0.807)	0.775 (95%CI:0.669‐0.882)	0.779 (95%CI:0.628‐0.929)	0.914 (95%CI:0.751‐1.077)	0.971 (95%CI:0.809‐1.133)
Serum marker at baseline						
PSA level, ng/mL						
>295	0.567 (95%CI:0.480‐0.654)	0.550 (95%CI:0.446‐0.655)	0.556 (95%CI: 0.421‐0.692)	0.531 (95%CI: 0.368‐0.694)	0.693 (95%CI:0.491‐0.894)	0.804 (95%CI: 0.581‐1.027)
≤295	0.596 (95%CI:0.510‐0.681)	0.578 (95%CI:0.479‐0.678)	0.668 (95%CI: 0.553‐0.782)	0.531 (95%CI:0.336‐0.726)	0.662 (95%CI: 0.426‐0.899)	0.758 (95%CI:0.496‐1.019)
Hb level, g/dL						
≤12	0.516 (95%CI:0.409‐0.623)	0.518 (95%CI:0.382‐0.654)	0.540 (95%CI: 0.346‐0.733)	0.628 (95%CI: 0.407‐0.850)	0.835 (95%CI:0.560‐1.110)	N/A
>12	0.613 (95%CI:0.539‐0.688)	0.588 (95%CI: 0.503‐0.673)	0.641 (95%CI:0.544‐0.739)	0.529 (95%CI: 0.389‐0.669)	0.666 (95%CI:0.497‐0.835)	0.774 (95%CI:0.587‐0.961)
ALP level, IU						
>350	0.478 (95%CI:0.386‐0.570)	0.489 (95%CI:0.378‐0.600)	0.493 (95%CI: 0.348‐0.639)	0.362 (95%CI: 0.192‐0.531)	0.475 (95%CI: 0.259‐0.691)	0.558 (95%CI: 0.313‐0.804)
≤350	0.663 (95%CI:0.583‐0.744)	0.621 (95%CI:0.527‐0.716)	0.701 (95%CI: 0.594‐0.808)	0.709 (95%CI: 0.566‐0.851)	0.884 (95%CI:0.719‐1.049)	1.013 (95%CI: 0.839‐1.186)
LDH level, IU						
>220	0.422 (95%CI:0.319‐0.525)	0.458 (95%CI:0.323‐0.592)	0.403 (95%CI: 0.223‐0.583)	0.297 (95%CI: 0.087‐0.506)	0.428 (95%CI: 0.132‐0.723)	N/A
≤220	0.662 (95%CI:0.588‐0.736)	0.612 (95%CI:0.527‐0.698)	0.693 (95%CI:0.595‐0.791)	0.649 (95%CI: 0.514‐0.783)	0.793 (95%CI: 0.639‐0.947)	0.902 (95%CI:0.736‐1.067)
Hormone therapy						
LHRH antagonists^a^						
Used	0.684 (95%CI:0.470‐0.826)	0.260 (95%CI:0.031‐0.593)	0.291 (95%CI:0.031‐0.645)	N/A	N/A	N/A
Not used	0.586 (95%CI:0.519‐0.647)	0.576 (95%CI:0.498‐0.645)	0.629 (95%CI:0.530‐0.713)	0.557 (95%CI: 0.423‐0.671)	0.710 (95%CI: 0.526‐0.833)	0.823 (95%CI: 0.572‐0.935)
Antiandrogen						
Used	0.579 (95%CI:0.512‐0.645)	0.574 (95%CI:0.497‐0.652)	0.628 (95%CI: 0.532‐0.725	0.574 (95%CI: 0.444‐0.703)	0.725 (95%CI:0.569‐0.882)	0.844 (95%CI: 0.672‐1.016)
Not used	0.637 (95%CI:0.487‐0.787)	0.512 (95%CI:0.293‐0.731)	0.558 (95%CI: 0.322‐0.793)	0.462 (95%CI: 0.171‐0.754)	0.600 (95%CI: 0.239‐0.962)	0.643 (95%CI: 0.260‐1.026)
Local treatment						
Yes	0.613 (95%CI:0.398‐0.827)	0.621 (95%CI:0.404‐0.838)	0.673 (95%CI: 0.441‐0.905)	0.713 (95%CI: 0.473‐0.952)	0.965 (95%CI: 0.695‐1.234)	N/A
No	0.587 (95%CI:0.523‐0.651)	0.565 (95%CI:0.487‐0.642)	0.611 (95%CI:0.515‐0.708)	0.542 (95%CI:0.414‐0.670)	0.685 (95%CI:0.531‐0.8400)	0.775 (95%CI: 0.606‐0.943)

Abbreviations: ALP, alkaline phosphatase; BMI, body mass index; CI, confidence interval; CS, conditional survival; ECOG‐PS, Eastern Cooperative Oncology Group Performance Status; EOD, extent of bone disease; Hb, hemoglobin; LDH, lactate dehydrogenase; LHRH, Luteinizing hormone‐releasing hormone; PSA, prostate‐specific antigen

a 95%CI estimated based on log‐transformation

**Figure 3:**
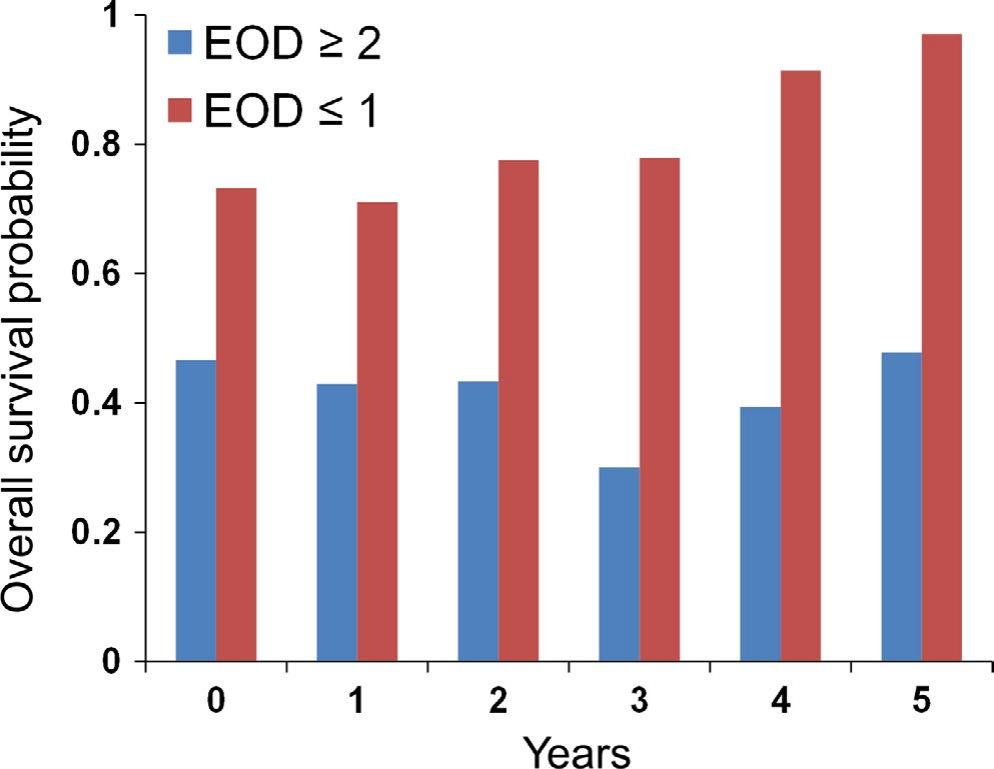
Conditional 5‐y net overall survival (OS) rates relative to the baseline rate. The bars indicate the conditional 5‐y net OS rates for patients with metastatic hormone‐naive prostate cancer initially treated with androgen deprivation therapy

**Figure 4:**
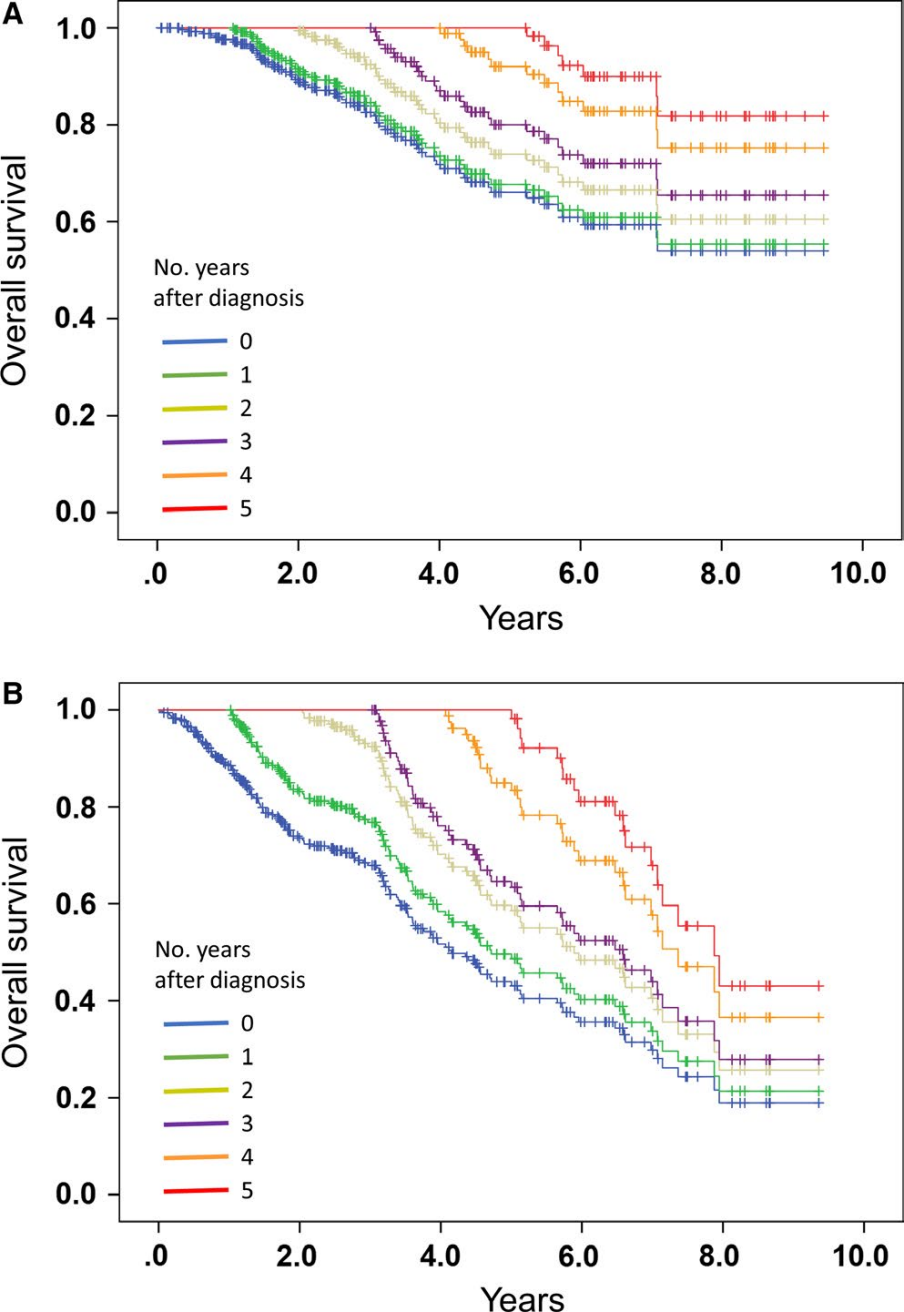
Conditional overall survival curves stratified by the bone metastasis extent of disease (EOD) score. (A): EOD ≤1. (B): EOD ≥2

Next, we used multivariate analyses to assess the changing hazard ratios for conditional 5‐year net CSS and OS rates for up to 5 years (Table 3 and Table S4). Several variables were identified as prognostic factors for CSS and/or OS at baseline, including BMI ≤18.5kg/m^2^, ECOG‐PS ≥1, the presence of lymph node metastasis, high PSA levels at baseline, low Hb level at baseline, and high LDH level at baseline; however, after the 2‐year time point, these variables were no longer independent prognostic factors for CSS and OS (Table 3 and Table S4). Only EOD ≥2 remained a prognostic factor for CSS and OS for each year of survival (except for CSS in the cohort that survived 2 years). At baseline, the conditional 5‐year net CSS and OS rates for the patients with EOD ≥2 were 0.541 and 0.466, respectively; for the patients who survived 5 years, they were 0.647 and 0.478, respectively (Table 2, Figure 3, Table S3, and Figure S3). Kaplan‐Meier curves for conditional CSS and OS based on the EOD score are shown in Figure 4 and Figure [Link], [Link]. The OS hazard ratios associated with EOD ≥2 in the conditional versions of the Cox regression models for 1,2,3,4, and 5 years were 1.83, 1.74, 2.13, 2.57, and 3.82, respectively (Table 3). Biopsy Gleason score ≥9 remained an independent prognostic factor for CSS or OS at 3 and 2 years after the diagnosis, respectively, but these were subsequently no longer statistically significant. These results suggested that the prognosis for patients with EOD ≥2 remained poor over time and that a higher number of bone metastases remained a durable prognostic factor for CSS and OS at all survival time points up to 5 years.

**Table 3:** Proportional hazard ratios for conditional 5‐y net overall survival in multivariate Cox regression analyses for the prediction of overall mortality

	Baseline (n = 605)	1 y (n = 488)	2 y (n = 341)	3 y (n = 249)	4 y (n = 165)	5 y (n = 112)
BMI < 18.5 kg/m^2^ *						
*P*‐value	.007	.041	.085	.177	.746	.458
HR	2.05	1.85	2.03	1.89	0.72	0.43
95%CI	1.21‐3.45	1.03‐3.32	0.91‐4.55	0.75‐4.80	0.10‐5.24	0.05‐3.96
BMI 18.5‐24.9 kg/m^2^ *						
*P*‐value	.780	.927	.637	.901	.311	.830
HR	1.06	0.98	1.14	1.04	1.55	1.13
95%CI	0.71‐1.57	0.64‐1.50	0.66‐1.97	0.56‐1.91	0.67‐3.59	0.38‐3.39
ECOG‐PS ≥1						
*P*‐value	.017	.096	.146	.346	.786	.079
HR	1.43	1.32	1.35	1.25	0.91	0.43
95%CI	1.07‐1.93	0.95‐1.83	0.90‐2.03	0.79‐1.98	0.44‐1.85	0.17‐1.10
Biopsy Gleason score ≥9						
*P*‐value	.001	.002	.009	.088	.198	.576
HR	1.65	1.72	1.76	1.51	1.55	1.27
95%CI	1.22‐2.24	1.23‐2.41	1.15‐2.67	0.940‐2.41	0.80‐3.00	0.54‐2.98
EOD score ≥2						
*P*‐value	<.0001	.001	.015	.004	.008	.005
HR	2.07	1.83	1.74	2.13	2.57	3.82
95%CI	1.48‐2.89	1.27‐2.62	1.11‐2.71	1.28‐3.53	1.28‐5.18	1.50‐9.71
PSA level >295 ng/mL						
*P*‐value	.013	.191	.586	.604	.942	.697
HR	0.68	0.79	0.89	0.88	0.97	1.20
95%CI	0.50‐0.92	0.56‐1.12	0.57‐1.37	0.53‐1.45	0.48‐1.96	0.49‐2.93
Hb level ≤12 g/dL						
*P*‐value	.008	.025	.542	.825	.755	.165
HR	1.57	1.55	1.17	1.07	1.14	2.00
95%CI	1.13‐2.19	1.06‐2.26	0.71‐1.94	0.59‐1.93	0.50‐2.58	0.75‐5.31
LDH level >220 IU						
*P*‐value	.001	.079	.090	.067	.427	.621
HR	1.75	1.37	1.47	1.60	1.32	1.25
95%CI	1.27‐2.40	0.97‐1.94	0.94‐2.29	0.97‐2.65	0.67‐2.60	0.51‐3.05

Abbreviations: ALP, alkaline phosphatase; BMI, body mass index; 95%CI, 95% confidence interval; ECOG‐PS, Eastern Cooperative Oncology Group Performance Status; EOD, extent of bone disease; Hb, hemoglobin; HR, hazard ratio; LDH, lactate dehydrogenase; PSA, prostate‐specific antigen

* Compared to BMI ≥25 kg/m^2^

## 4 DISCUSSION

This study was the first study to investigate conditional net survival in prostate cancer patients using an unbiased novel estimator. We showed that, after the baseline survival estimation, the conditional net CSS and OS rates gradually increased with time. Furthermore, the significance of prognostic factors for the patients with mHNPC changed over time after ADT. Only EOD ≥2 remained an independent factor for CSS and OS, whereas other well‐known prognostic factors had lost their statistical significance as prognostic factors by 5 years after the administration of ADT therapy.

In general, conditional survival gains are limited for patients at low risk, whereas the relationship is stronger for patients with adverse prognostic features.^29^, ^30^ In a study to assess population‐based 5‐year conditional survival for various cancers, Janssen‐Heijnen et al reported that conditional 5‐year relative survival in patients with prostate cancer decreased from 89% at diagnosis to 81% at 6 years after diagnosis.^31^ Conditional relative survival analyses for a large number of cancers based on the Canadian Cancer Registry showed that the conditional survival in prostate cancer did not change 5 years after diagnosis.^32^ In the largest population‐based study for conditional survival, which included 204 472 patients with prostate cancer in the United States, Merrill et al reported an increase in CSS after survival for five years, with the probability of remaining disease‐free up to year 5 increasing from 33.1% to 55.9%.^33^ Thus, there have been inconsistent results reported for conditional survival rates based on cancer registries in patients with prostate cancer. Further application of unbiased estimators is needed to assess conditional survival and to perform conditional survival analyses targeted at this specific subgroup population to provide more precise information for the patients.

Newly diagnosed metastatic prostate cancer is not a curative disease, and a recent study that evaluated survival in patients with stage IV prostate cancer registered in the SEER database between 2004 to 2010 showed that the 5‐year OS was 22% to 67.4%,^8^, ^9^ providing rather pessimistic information for patients with mHNPC. Only one study has specifically reported conditional survival in patients with stage IV prostate cancer. Muralidhar et al assessed conditional cancer‐specific mortality in 41 022 M1 patients registered in the SEER database and reported that 5‐year prostate cancer‐specific mortality improved from 57.2% at diagnosis to 41.1% at 5 years, 28.8% at 10 years, and 20.8% at 15 years.^16^ Although that study had some limitations, including being based on an old database with patients diagnosed between 1973 and 2011, as well as a lack of detailed background other than age, race, income, married status, and Gleason grading, it showed that the risk of death decreased overtime in patient with advanced stage prostate cancer. This study, which is the first study to assess the conditional net survival specific to metastatic prostate cancer using the unbiased novel Pohar Perme estimator, demonstrated that 5‐year CSS and OS rates significantly increased from 65.5% and 58.2% to 90.6% and 81.1%, respectively. Taken together, conditional survival estimates may provide more appropriate information for patients with mHNPC.

The stratification of conditional survival estimates by prognostic factors provides more relevant clinical information and better estimates of individual patient prognosis.^32^, ^34^ In line with previous studies that reported baseline risk factors for mHNPC,^10^, ^35^, ^36^ our multivariate analysis confirmed the impact of potential baseline prognostic factors for CSS and OS in mHNPC. Poorer CSS and OS rates at the time of diagnosis were observed for subgroups based on low Hb, high LDH level, BMI ≤18.5kg/m^2^, ECOG‐PS ≥1, and the presence of lymph node metastasis; however, these differences diminished in the following years, suggesting that the prognostic significance of these factors decreases as time elapses after diagnosis. In contrast, the number of bone metastases remained a significant risk factor even after 5 years of follow‐up. These findings could be used to drive a more evidence‐based strategy for post‐treatment follow‐up scheduling that is based on the patient's actual current risk rather than simply on baseline probabilities.

Bone metastatic tumor burden is one of the most influential prognostic markers in patients with mHNPC.^10^, ^37^ In large randomized trials that showed the benefit of upfront therapy using docetaxel and abiraterone acetate with ADT in patients with mHNPC, the number of bone metastases was one of the specific factors used for dichotomizing risk groups for prognosis.^6^, ^38^ A retrospective study that included 304 Japanese patients with treatment‐naïve castration‐sensitive prostate cancer reported that EOD ≥2 was an independent risk factor, and EOD ≥2 was one of four risk factors used in the study to develop three risk categories.^37^ Consistent with these results, this study showed that EOD ≥2 was an independent prognostic factor and showed for the first time that the number of bone metastases continued to influence the CSS and OS of patients with mHNPC over time. The conditional survival rate remained low for patients with EOD ≥2 but generally increased for the entire cohort and the other subgroups. These findings give the intriguing possibility of developing more personalized treatment and/or follow‐up for individual patients with mHNPC and of providing these patients with more accurate information about their prognosis. However, conditional survival rates have not yet been established for patients treated with upfront abiraterone acetate and docetaxel with ADT, which has become a novel standard treatment for high‐risk and high‐volume mHNPC. Among the other prognostic variables considered in the multivariate analysis, biopsy Gleason score ≥9 remained a statistically significant independent prognostic factor for CSS and OS until 3 years and 2 years, respectively. The results of the LATITUDE trial suggested that three risk factors could be used as an indication for the upfront administration of abiraterone: biopsy Gleason score ≥8, the presence of three or more bone lesions, and/or the presence of measurable visceral metastases.^6^ Although our results do not provide a definitive assessment, they strongly support these two factors—the number of bone metastases and biopsy Gleason score—as being risk factors for CSS and OS in patients with newly diagnosed mHNPC. However, the present study did not reveal any impact of visceral metastasis on CSS and OS, perhaps because of the small number of patients compared with the numbers included in the previous trials (14.0%‐18%).^6^, ^39^

Our study had several limitations. First, the multicenter design resulted in heterogeneity of the patients' treatment and monitoring. Second, the study did not consider the impact of sequential treatments after the initial ADT. The sequential therapy given after development of CRPC was described in Table S5. Although there was no statistical association of year of diagnosis with CSS and OS in this study, sequential treatments following ADT failure may have played a role in the outcomes for some patients. The retrospective study design and short follow‐up duration were further limitations. Future studies with a longer follow‐up period and a validation dataset are warranted.

In conclusion, the conditional 5‐year net CSS and OS rates in patients with mHNPC gradually increased in the years following ADT treatment, implying that the risk of mortality decreased with increasing length of survival. The patients' risk profiles changed over time, but the EOD score remained an independent prognostic factor for CSS and OS after 5 years of follow‐up. Conditional net survival can play a role in clinical decision‐making and provides valuable information for cancer survivors.

## CONFLICT OF INTEREST

Tomonori Habuchi has acted as a paid consultant for Janssen and Sanofi for work performed outside the current study.

## AUTHOR CONTRIBUTIONS

Shintaro Narita: Data collection, statistical analysis, manuscript writing. Hiromi Sato, Shingo Hatakeyama, Masahiro Takahashi, Toshihiko Sakurai, Sadafumi Kawamura, Masanori Ishida, Senji Hoshi: Data collection. Toshiaki Kawaguchi, Shigeto Ishidoya, Jiro Shimoda, Koji Mitsuzuka, Kengo Nagashima: supervision. Kyoko Nomura: Statistical analysis, manuscript writing. Tatsuo Tochigi, Norihiko Tsuchiya, Chikara Ohyam, Yoichi Arai, Tomonori Habuchi: manuscript editing, supervision. All authors have read and approved of the final manuscript.
