# Supplementary figures and images for: Changes in conditional net survival and dynamic prognostic factors in patients with newly diagnosed metastatic prostate cancer initially treated with androgen deprivation therapy
Source: Cancer Med. 2019 Sep 11;8(15):6566–77. doi: 10.1002/cam4.2502 (PMC6825980; doi:10.1002/cam4.2502)

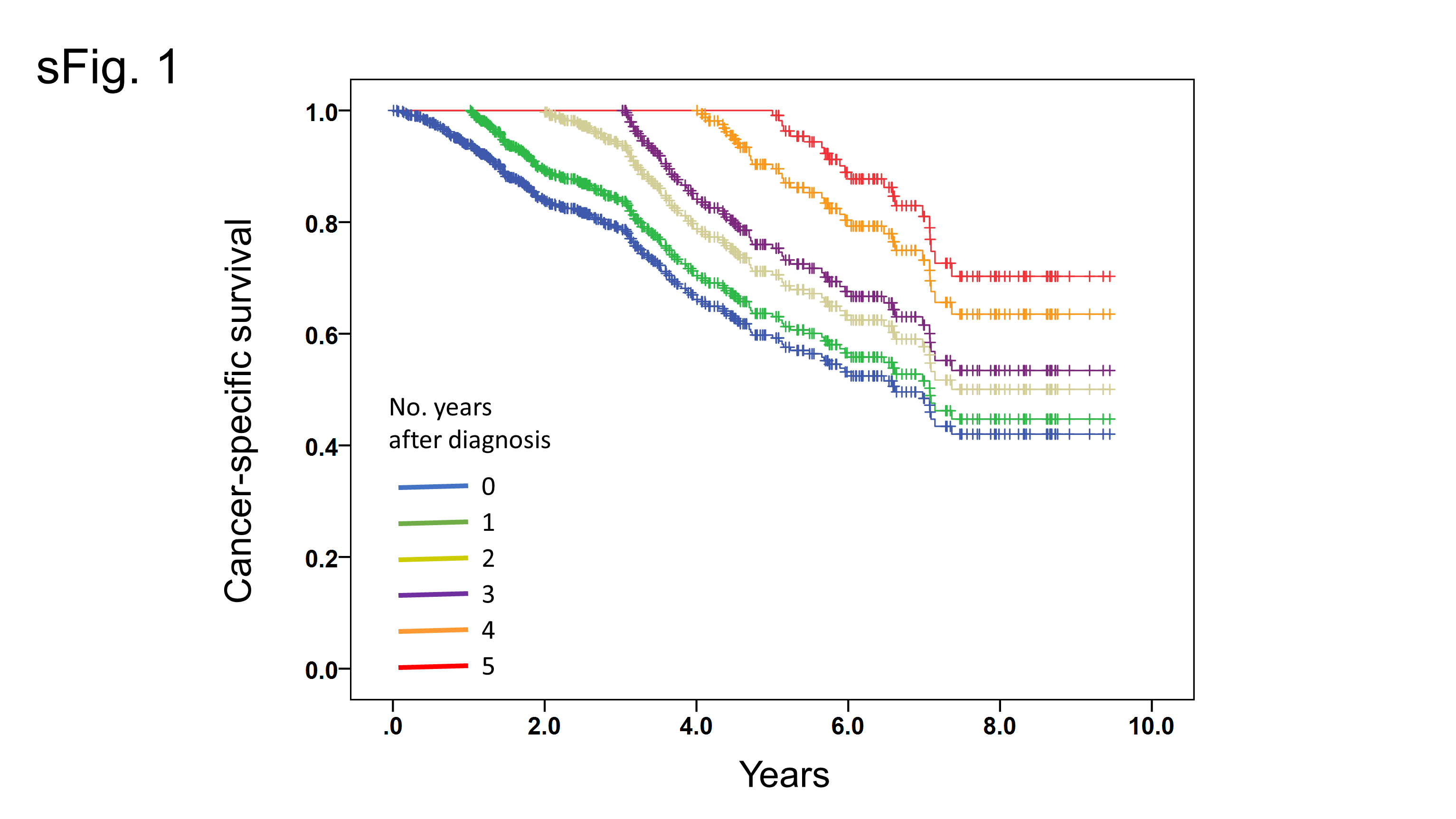

Supplement: Supplementary file 1 [file CAM4-8-6566-s001.TIF]

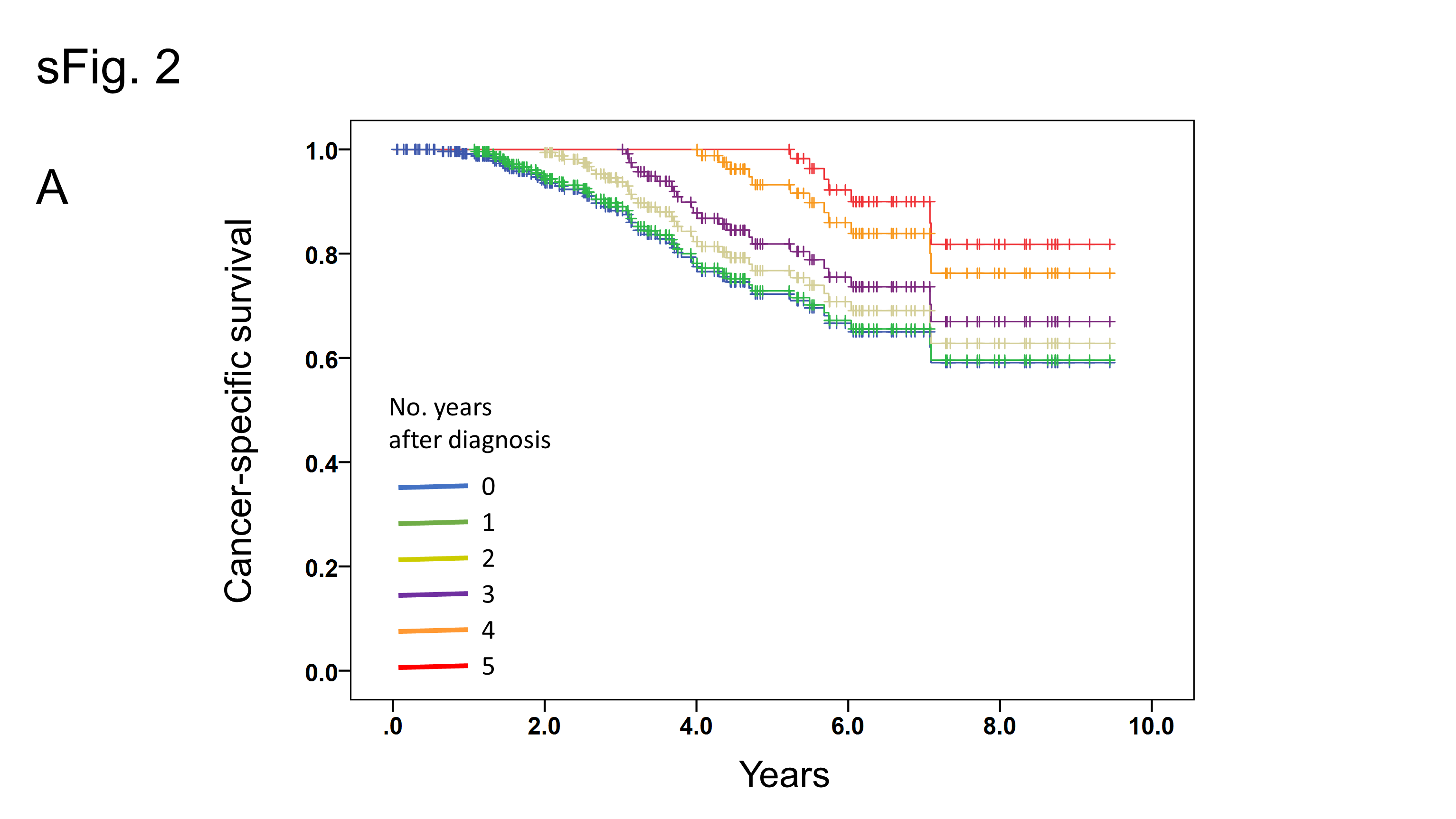

Supplement: Supplementary file 2 [file CAM4-8-6566-s002.TIF]

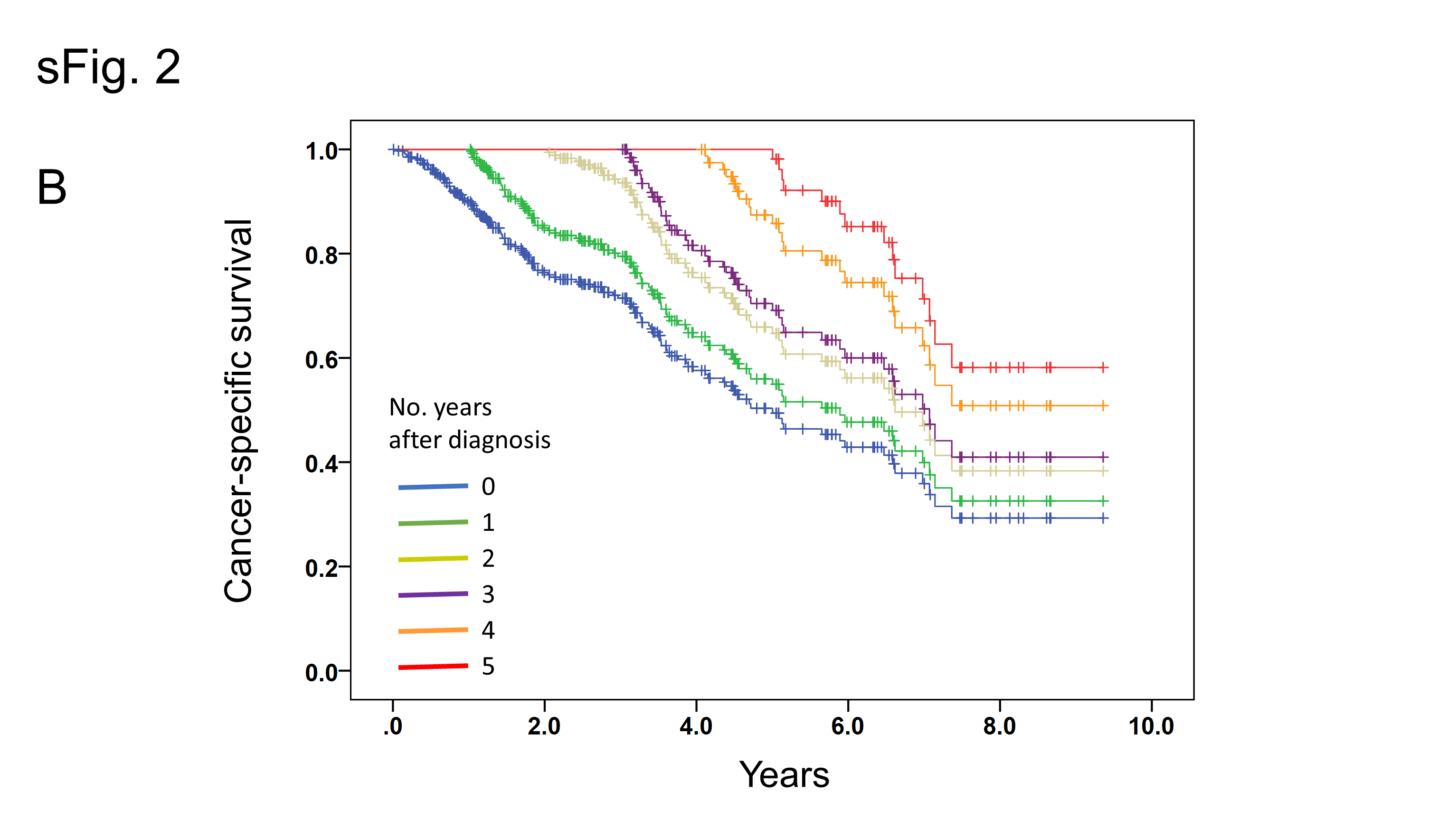

Supplement: Supplementary file 3 [file CAM4-8-6566-s003.TIF]

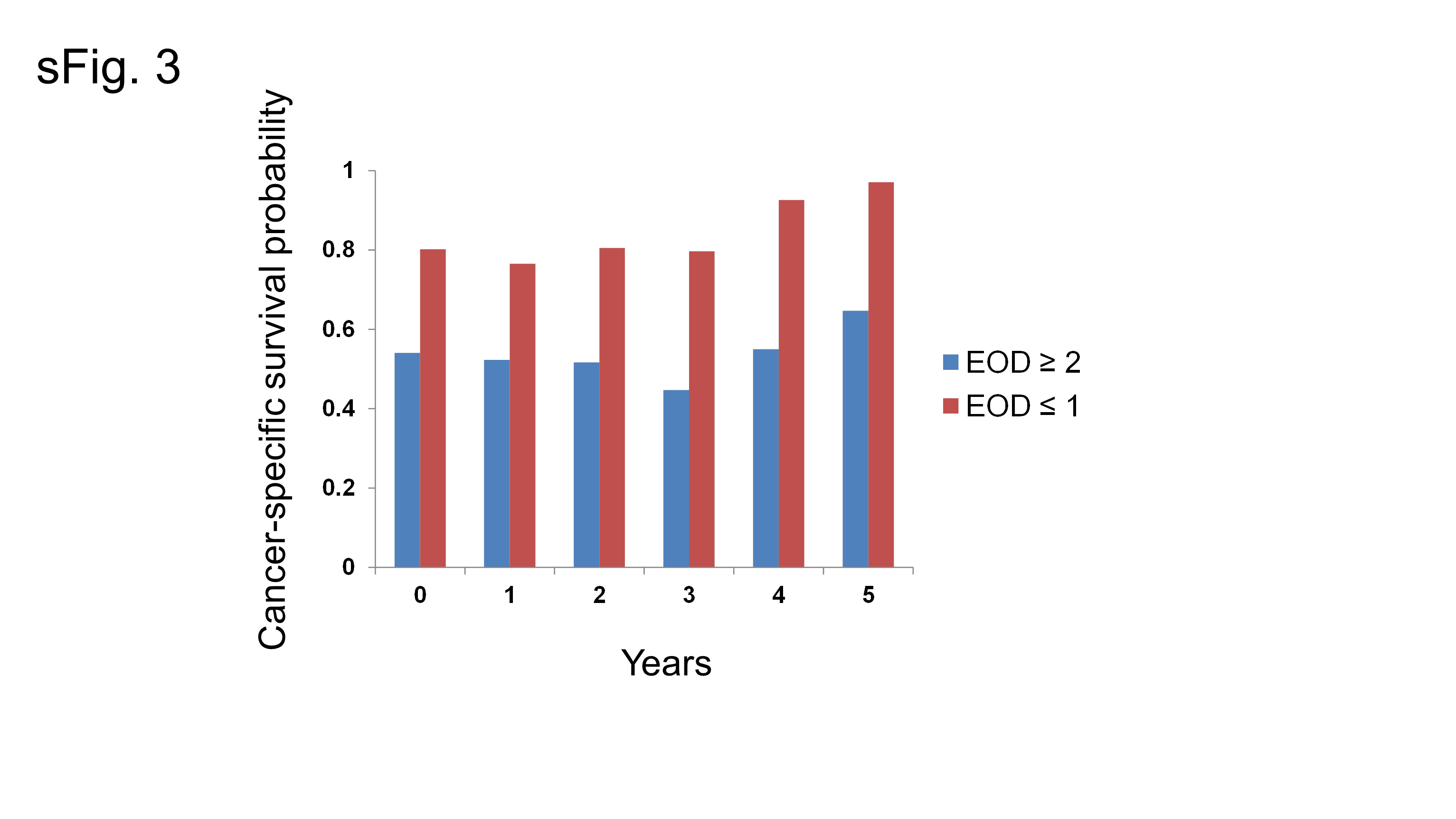

Supplement: Supplementary file 4 [file CAM4-8-6566-s004.TIF]
